# Cell biology of primary cell wall synthesis in plants

**DOI:** 10.1093/plcell/koab249

**Published:** 2021-10-06

**Authors:** Ying Gu, Carolyn G Rasmussen

**Affiliations:** Department of Biochemistry and Molecular Biology, Pennsylvania State University, University Park, Pennsylvania 16802; Department of Botany and Plant Sciences, Center for Plant Cell Biology, University of California, Riverside, California 92521

## Abstract

Building a complex structure such as the cell wall, with many individual parts that need to be assembled correctly from distinct sources within the cell, is a well-orchestrated process. Additional complexity is required to mediate dynamic responses to environmental and developmental cues. Enzymes, sugars, and other cell wall components are constantly and actively transported to and from the plasma membrane during diffuse growth. Cell wall components are transported in vesicles on cytoskeletal tracks composed of microtubules and actin filaments. Many of these components, and additional proteins, vesicles, and lipids are trafficked to and from the cell plate during cytokinesis. In this review, we first discuss how the cytoskeleton is initially organized to add new cell wall material or to build a new cell wall, focusing on similarities during these processes. Next, we discuss how polysaccharides and enzymes that build the cell wall are trafficked to the correct location by motor proteins and through other interactions with the cytoskeleton. Finally, we discuss some of the special features of newly formed cell walls generated during cytokinesis.

## Teaser

The cell wall is assembled via vesicle trafficking along cytoskeletal filaments during growth and division.

## Introduction

The study of structure and function of the cell wall began unintentionally in 1665 when Robert Hooke examined oak (*Quercus suber*) cork under a microscope. He coined the term “cell” to describe the smallest living biological structure based on its similarity to a monk’s room (cell). Since the cellular contents were gone, Hooke actually visualized the cell wall. Since then, modern cell wall research benefited from the characterization of structural components of the extracted cell wall (Harris et al., 2010). Even more recently, cell biology, including live-cell imaging, has been instrumental in advancing our understanding of transport and synthesis of cell wall components and their interaction with the cytoskeleton. Due to space limitations, this review focuses primarily on cytoskeleton-based trafficking of cell wall synthesis and modification enzymes during diffuse growth and cytokinesis. There are excellent reviews of cell wall synthesis and cell growth focused more on tip growing cells (Chebli et al., 2021), cell wall and cytoskeletal modifications during the plant immune response (Bacete et al., 2018; Li and Staiger, 2018), cell wall integrity sensing (Wolf et al., 2012), and secondary cell wall synthesis (Schneider et al., 2016; Meents et al., 2018; Coomey et al., 2020).

Cell division and expansion are two primary generators of plant biomass. As a major component of primary cell walls, cellulose is the most abundant biopolymer on the planet. In addition to global biomass, production and modification of the cell wall is vital to plants. Indeed, around 10% of some plant genomes are invested in cell wall formation and modification (Carpita and McCann, 2008) with an additional hundreds to thousands of genes involved in regulating the cytoskeleton and vesicle trafficking.

Two different stages of cell wall formation are discussed: the de novo construction of the cell wall during cytokinesis and the addition of new cell wall material into already extant cell walls during diffuse growth. In both cases, vesicles containing cell wall materials are trafficked on the cytoskeleton by motor proteins. Vesicles typically originate from the Golgi and are trafficked on the cytoskeleton often through the trans Golgi network (TGN) to and from the plasma membrane and the cell plate. Therefore, properly organized microtubules and microfilaments are critical for the positioning and construction of cell wall components. For example, the rapid and properly positioned addition of cellulose during diffuse growth relies on well-organized microtubules due to direct interactions between microtubules, microtubule linking proteins, and enzymes that generate cell wall polymers called cellulose synthases.

The de novo cell wall is initiated by a conserved land–plant specific structure called the phragmoplast during division (Jürgens, 2005; Buschmann and Zachgo, 2016). The main purpose of the phragmoplast is to generate a new cell wall after separation of duplicated DNA during cytokinesis (Smertenko et al., 2017; Lee and Liu, 2019). An intermediate form of the new cell wall, called the cell plate, is constructed prior to the completion of cytokinesis. Generation and expansion of the cell plate are accompanied by coordinated vesicle trafficking and fusion as well as changes in cell wall composition (Sinclair et al., 2018). The phragmoplast, which contains short actin filaments, forms as an antiparallel array of microtubules, with their plus-ends facing the middle of the cell (Smertenko et al., 2018). The microtubules within the phragmoplast are assembled from spindle microtubules followed by phragmoplast expansion mediated by microtubule-dependent microtubule nucleation (Smertenko et al., 2011; Murata et al., 2013). These microtubules are then disassembled as the cell plate expands (Sasabe and Machida, 2012). The phragmoplast contacts the cortex in a defined location known as the division site or cell plate fusion site (Smertenko et al., 2017; Rasmussen and Bellinger, 2018; Livanos and Müller, 2019). Similar to diffuse growth, cytokinesis depends heavily on proper organization of the cytoskeleton. Therefore, microtubules and microfilaments and proteins that affect cytoskeletal dynamics and organization are discussed in the next section. Specific proteins mentioned in the text can be found in Supplemental Data Set S1.

## Factors that organize microtubules and microfilaments at the cell cortex during diffuse growth

Microtubules and actin microfilaments serve as tracks along which motor proteins move cargo, including cell wall components. Factors that mediate microtubule and microfilament positioning play a critical yet sometimes indirect role in how the cell wall is modified, expanded, or generated. Here, we discuss how microtubule and microfilament orientation at the cell cortex are influenced by cell shape and mechanical forces.

Observation of cortical microtubules oriented parallel to the long axis of elliptical or rectangular microchambers supports the hypothesis that cell geometry determines microtubule orientation (Lagomarsino et al., 2007). Microtubule organization was recently empirically tested in protoplasts, which are single cells with enzymatically removed cell walls. When protoplasts are confined in rectangular wells, microtubules align along the long axis, leading to the hypothesis that default microtubule orientation is longitudinal (Durand-Smet et al., 2020). In contrast, in planta, microtubules are often perpendicular to the long axis in many cell types (Hejnowicz, 2000; Hamant et al., 2008; Jacques et al., 2013; Sampathkumar et al., 2014). Therefore, microtubules must be re-oriented: a weak directional cue is sufficient to induce microtubule orientation switching from longitudinal to transverse in silico (Mirabet et al., 2018). Mechanical stress is proposed as one of the directional cues. The simplest model states that microtubules respond to mechanical stress and then guide the orientation of cellulose microfibrils along the direction of maximal tension (Green, 1964). In elongating cells, the growth direction is perpendicular, while microtubules align parallel to the predicted maximal tensile stress direction (Fischer and Schopfer, 1997, 1998; Baskin et al., 1999; Hejnowicz et al., 2000; Sugimoto et al., 2001; Burian et al., 2013). When the stress pattern is changed by laser ablation of nearby cells, microtubules become circumferential in meristems, cotyledons, and hypocotyls (Hamant et al., 2008; Sampathkumar et al., 2014; Robinson and Kuhlemeier, 2018). To further test the microtubule orienting mechanism, spherical protoplasts were confined in rectangular wells in solutions of different osmolarity. The tension induced by imposed reduction in osmolarity bypasses the geometrical cues to orient microtubules along predicted maximal tension after 2 h (Colin et al., 2020). It is unlikely that plant cells undergo similar osmotic changes and it remains to be determined how the change in osmotic potential translates into change in tensile stress. Microtubules may respond to the changes in tensile stress directly, but most likely multiple other proteins are required. Future research is needed to test whether microtubules, their regulators, or other factors are involved in sensing maximal tension.

Actin has vital roles in cell elongation and vesicle trafficking in tip growing cells such as root hairs and pollen tubes, beautifully reviewed here (Szymanski and Staiger, 2018). The importance of actin during diffuse growth and phragmoplast assembly is clear, but the underlying mechanisms are still unknown. Actin filament alignment becomes more parallel and ordered in elongating Arabidopsis (*Arabidopsis thaliana*) root cells, but the significance is thus far unknown (Dyachok et al., 2011; Arieti and Staiger, 2020). Disruption of the actin cytoskeleton disrupts Golgi movement and influences cellulose synthase complex (CSC) delivery to the plasma membrane (discussed in the “The assembly and trafficking of cellulose synthase complex” section) (Gutierrez et al., 2009; Sampathkumar et al., 2013). Actin may also mediate delivery of noncellulosic polysaccharides due to cell adhesion defects seen in actin and actin regulatory mutants and alteration in wall components by latrunculin B treatment (El-Din El-Assal et al., 2004; Chen et al., 2007; Dyachok et al., 2008). Actin plays different roles during different stages of phragmoplast assembly. Phragmoplast assembly was delayed when the actin-monomer stabilizing protein profilin was microinjected (Valster et al., 1997). In contrast, the transport of NACK1 kinesin (discussed in the “Plus-end-directed kinesins” section) to the phragmoplast midline during early phragmoplast development is sped up by disrupting actin filaments with latrunculin B (Maeda et al., 2020).

Actin motor proteins, myosins, also influence diffuse and polarized growth. There are 13 myosin XIs in Arabidopsis with often redundant functions. Myosin double, triple, and quadruple mutants have reduced cell expansion as do cells treated with a myosin ATPase inhibitor (Samaj et al., 2000; Peremyslov et al., 2008; Peremyslov et al., 2010; Madison et al., 2015). In moss, Myosin XI interacts with RabE and localizes to the phragmoplast midline. The interaction between RabE and myosin XI is critical for polarized growth (Sun et al., 2018; Orr et al., 2020). Reduction in the plant-specific Myosin VIII activity also slows cell expansion in moss (*Physcomitrium* (*Physcomitrella*) *patens*; Wu et al., 2011). Myosin contribution to diffuse growth may be due to its role in Golgi movement within the cell, leading to well-distributed cellulose synthase deposition.

Actin regulatory proteins called formins have demonstrated roles in polarized and diffuse growth (e.g. VidaLi et al., 2009; Cheung et al., 2010; Zhang et al., 2011b; Liu et al., 2018, 2021; Kollárová et al., 2021) but their roles in cytokinesis are less clear. Arabidopsis FORMIN HOMOLOGY5 (FH5) localizes to the cell plate and promotes actin filament formation in vitro. *fh5* mutants have reduced endosperm cellularization, suggesting slower cytokinesis (Ingouff et al., 2005). Additionally, FH5 interacts with the membrane-tubulation protein SH3P2 which has an essential role in cell plate formation (Baquero Forero and Cvrčková, 2019). Rice (*Oryza sativa*) FH5 and Arabidopsis FH4 bind both microtubules and actin, indicating crosstalk between microtubules and the actin cytoskeleton (Deeks et al., 2010; Yang et al., 2011). Lesions in rice *FH5* result in compromised longitudinal actin cables but they do not affect the organization of microtubules. However, the short and swollen internode cells of the *fh5 (bui1*) mutant indicate defects in diffuse growth (Yang et al., 2011; Zhang et al., 2011b). Similar to FH4/FH5, Arabidopsis FORMIN 14 co-localizes with microtubules, binds actin and microtubules in vitro, and is important for cytokinesis during male meiocyte development (Li et al., 2010; Du et al., 2021).

Several valuable approaches targeting groups of formins have also been used to clarify their functions. When all of the class 1 formins were silenced with RNAi in moss, tip growth was unaffected but plants were smaller with fewer cells, suggesting delayed cytokinesis. No stubs or multinucleate cells were observed (VidaLi et al., 2009). Despite often non-obvious roles in cytokinesis, formins sometimes localize to the phragmoplast or phragmoplast midline (e.g. Li et al., 2010; Wu and Bezanilla, 2014; van Gisbergen et al., 2020). The small molecule formin inhibitor SMIFH2, which sidesteps potentially redundant formin function, was used to understand how formins contribute to mitosis and cytokinesis (Zhang et al., 2021). SMIFH2 treatment reduces formin recruitment to the phragmoplast, slows telophase progression, and generates wavy cell plates. Treatment with SMIFH2 also reduces EB1 comet length, and alters turnover of vesicle fusion proteins DYNAMIN RELATED PROTEIN 1A (DRP1A) and KNOLLE (Zhang et al., 2021). This powerful tool might also inhibit myosins in plants as it does in other organisms (Nishimura et al., 2020). Although SMIFH2 concentrations used in tobacco were 10–20-fold lower than those reported in Nishimura et al. (2020), it remains to be conclusively determined whether SMIFH2-induced cytokinesis defects are attributable to disruption solely of formins. Combinations of loss-of-function formin mutants may provide conclusive evidence about their roles in growth and cytokinesis.

## Factors that organize microtubules and microfilaments at the cell cortex during telophase in preparation for cytokinesis

**Figure 1:**
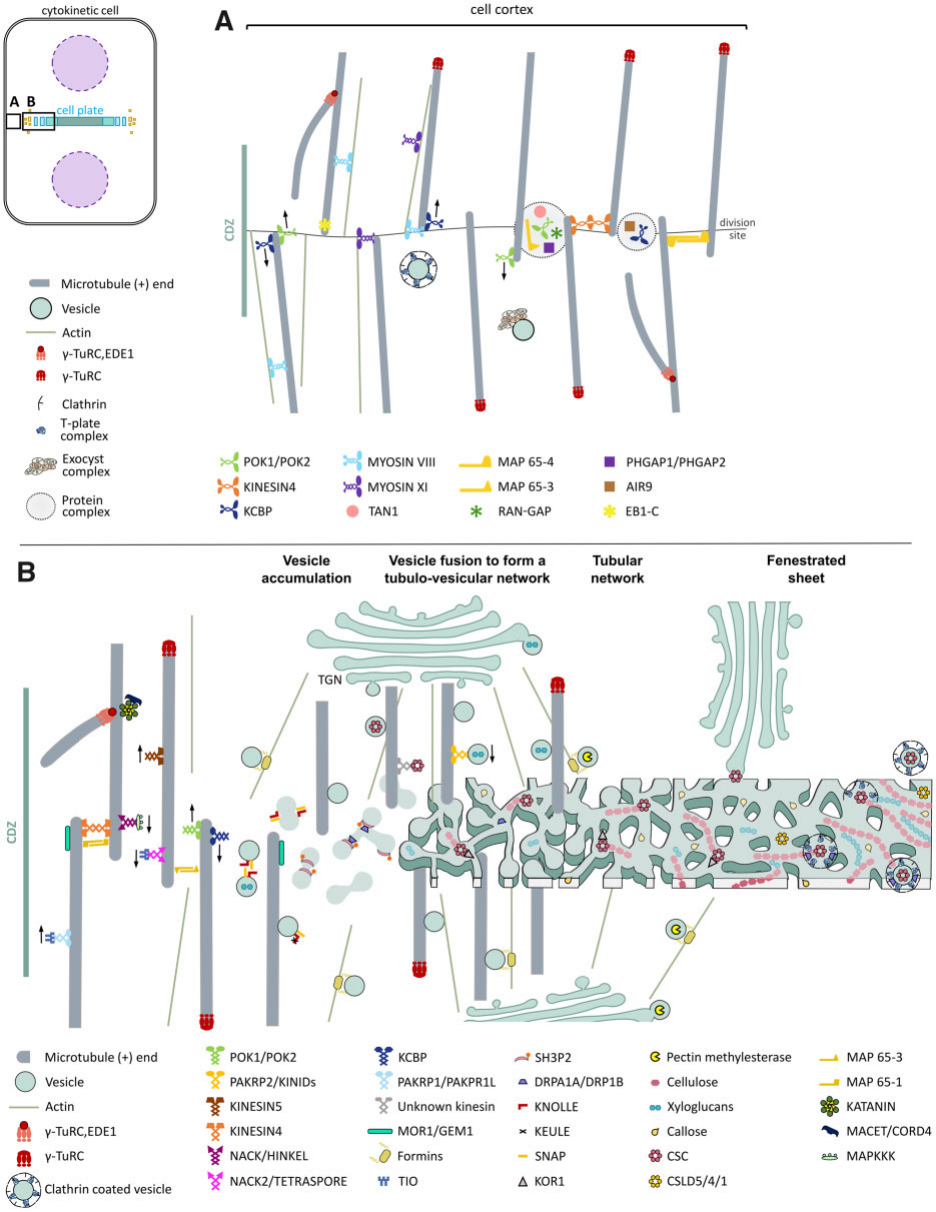
Model of land-plant cytokinesis. A, The cell cortex underlying the plasma membrane before it comes into contact with the phragmoplast. B, Phragmoplast, membrane, and cell wall structures that form the cell plate from left to right: vesicle accumulation, vesicle fusion to form a tubulo-vesicular network, a tubular network, and fenestrated sheet.

During telophase, but before the phragmoplast reaches the cell cortex, actin filaments and cortical telophase microtubules accumulate at the cell cortex (Figure 1, A; Schmit and Lambert, 1988; Liu and Palevitz, 1992; Panteris et al., 1995; Wu and Bezanilla, 2014). The microtubules nucleate from cortex-localized gamma tubulin and gamma tubulin ring complex structures and/or from the nuclear envelope (McDonald et al., 1993; Panteris et al., 1995; Van Damme, 2009; Kong et al., 2010). What microtubules and microfilaments do at the cell cortex during this stage is not well understood, but recent data suggest that they may be organized by interactions with proteins that localize to the division site (described in the “Proteins that localize to the division site before the phragmoplast reaches the cortex” section). Cortical telophase microtubules interact with TANGLED1 (TAN1) and other division site-localized proteins through transient microtubule plus-end stabilization, leading to their perpendicular orientation, similar to the orientation of the phragmoplast itself (Bellinger et al., 2021). Microtubule plus-end capture is consistent with the in vitro role of TAN1 angle-independent microtubule crosslinking and bundling (Martinez et al., 2020). Cortical telophase microtubules are added into the phragmoplast as it approaches the cell cortex. Further, if many cortical telophase microtubules accumulate on one side of the phragmoplast, they incorporate by parallel bundling into phragmoplast and then phragmoplast expansion along the cortex moves toward the accumulated cortical telophase microtubules. Therefore, cortical telophase microtubules fine tune the position of the phragmoplast to the division site, but it is still unknown how microtubule-binding proteins at the division site other than TAN1 interact with cortical telophase microtubules (Bellinger et al., 2021).

## Proteins that localize to the division site before the phragmoplast reaches the cortex

Proteins that accumulate at the division site before the phragmoplast contacts the cell cortex often have essential roles in cytokinesis or phragmoplast positioning. These include the microtubule-crosslinking protein TAN1, the microtubule binding kinesins PHRAGMOPLAST ORIENTING KINESIN (POK1) and POK2 (discussed in the “Plus-end-directed kinesins” section), PLECKSTRIN HOMOLOGY ROP-GAPs (PH-GAPs), the actin and microtubule binding myosin MYOSIN VIII (discussed in the “Phragmoplast cortex interactions” section), MYOSINXI (1, 2, K) (discussed in the next paragraph), and several IQ67 Domain (IQD) proteins. TAN1 is critical for phragmoplast positioning in maize, and bundles microtubules in vitro (Cleary and Smith, 1998; Walker et al., 2007; Martinez et al., 2017, 2020; Smith et al. 1996, 2001). In Arabidopsis, *tan1* mutants do not have aberrant phenotypes (Walker et al., 2007), unless combined with a mutant in *AUXIN INDUCED IN ROOT CULTURES9* (*AIR9*), which itself does not have a phenotype deviating from the wild-type situation (Buschmann et al., 2015; Mir et al., 2018)*.* In contrast, *tan1 air9* double mutants have division plane defects and short roots (Mir et al., 2018). Mitotic expression of TAN1 restores wild-type growth to the *tan1 air9* double mutant (Mills et al., 2021). The AIR9 protein localizes to the phragmoplast and accumulates at the division site after the phragmoplast contacts the cortex (Buschmann et al., 2006, 2015). Two PH-GAPs that interact with POK1 are redundantly required for division plane positioning and accumulate at the division site after metaphase (Stöckle et al., 2016). Finally, three related IQ67 DOMAIN (IQD6, 7, 8) proteins localize to the division site, interact with POK1, and have a role in division plane establishment (Kumari et al., 2021). Assessing localization of these division site proteins in various mutants will provide useful information about how distinct factors establish and maintain the division site until cytokinesis is complete.

Triple *myosin XI* (*xi-1 xi-2 xi-k*) mutants have defects in division plane orientation that are rescued by a MYOSIN XI-YFP that localizes to mitotic structures and also the division site. MYOSIN XIs (1, 2, K) promote division plane positioning but when they function during mitosis is currently unknown (Abu-Abied et al., 2018). OPAQUE1, a myosin XI similar to Arabidopsis XI-I, is essential for proper ER organization in maize kernels (Wang et al., 2012) OPAQUE1 is required for phragmoplast guidance in asymmetric divisions and localizes to the phragmoplast midline (Nan et al., 2021). Interestingly, OPAQUE1 interacts with other myosins, actin-binding proteins, and maize POKs. One hypothesis is that OPAQUE1-POK interactions promote phragmoplast guidance to the division site, although this remains to be experimentally tested (Nan et al., 2021).

Several other proteins also localize to the division site and are vital for cytokinesis but their functions in division plane positioning are unclear. The monomeric GTPase RAN GTPase ACTIVATING PROTEINS (RAN-GAPs) localize to the division site and the nuclear envelope, and their knockdown causes severe cytokinesis and growth defects preventing analysis of division plane positioning (Xu et al., 2008). Interestingly, RAN-GAP in onion (*Allium cepa*) localizes only transiently to the division site, indicating variation in RAN-GAP division site localization may vary across species (Yabuuchi et al., 2015). A microtubule associated protein (MAP) MAP65-4, described more in the “Microtubule bundling proteins with critical roles in diffuse growth and cytokinesis” section, localizes to the division site in addition to the phragmoplast midline, but what it does at the division site is not yet known (Li et al., 2017a). The exocyst complex proteins SECRETORY3 (SEC3), SEC6, SEC5 (and SEC5 paralogs) localize to the cell cortex before the phragmoplast touches the cortex as a wide band that reorients toward the tilting phragmoplast in *Physcomitrium patens*, but what they do at the cortex remains unknown (Tang et al., 2019).

## Phragmoplast cortex interactions

When the phragmoplast reaches the cortex, interactions between the phragmoplast and cortex-localized microtubules and actin filaments guide the phragmoplast precisely to the division site. These interactions are mediated by division site-localized proteins, such as the microtubule and microfilament binding protein MYOSIN VIII in moss (Wu and Bezanilla, 2014). F-actin, Myosin, and cell cortex interactions play an important role in the last slow step of phragmoplast expansion to meet with the mother cell plasma membrane: disruption leads to defects in final phragmoplast-mediated contact with the cell cortex (Chua et al., 2002; Molchan et al., 2002; Gilliland et al., 2003; van Oostende-Triplet et al., 2017). Interestingly, when the phragmoplast is displaced by centrifugation, microns long actin filaments contact the division site. The phragmoplast is subsequently recruited back to the division site, suggesting long distance actindivision site interactions (Arima et al., 2018). Much remains to be learned about how actin and microtubules, and the proteins that crosslink them, function together to guide the phragmoplast to the division site and to complete cytokinesis.

Microtubule regulators such as nucleators, severing proteins, end-binding proteins, and cross-linkers are vital for proper cellular elongation and cytokinesis (Hamada, 2014). Their roles can often be clarified using genetic and biochemical analyses. While many microtubule-associated proteins are essential for both diffuse growth and cytokinesis, others have more specific roles.

## Microtubule nucleators

Microtubule nucleators such as gamma tubulin, and the gamma-tubulin ring complex are essential for diffuse growth and cytokinesis (Liu et al., 1993; Shimamura et al., 2004; Murata et al., 2005; Pastuglia et al., 2006; Hotta et al., 2007; Seltzer et al., 2007; Ambrose and Wasteneys, 2014). Unlike animal cells, microtubules nucleate from the plasma membrane, the nucleus, or from preexisting microtubules (Murata et al., 2005; Hotta et al., 2007; Kong et al., 2010; Lee and Liu, 2019). Nucleation from preexisting microtubules depends on the AUGMIN complex and two MOZART1 homolog proteins GAMMA-TUBULIN COMPLEX3 INTERACTING PROTEIN1 (GIP1) and GIP2 to recruit gamma tubulin ring complexes (Ho et al., 2011b; Janski et al., 2012; Hotta et al., 2012; Nakamura et al., 2012; Nakaoka et al., 2012; Murata et al., 2013; Oh et al., 2016; Lee et al., 2017; Lee and Liu, 2019).

During cytokinesis, the phragmoplast expands by addition of new microtubules nucleating on the phragmoplast leading edge (Figure 1, B). Therefore, microtubule-dependent nucleation is critical for phragmoplast expansion (Smertenko et al., 2011; Murata et al., 2013). ENDOSPERM DEFECTIVE1 (EDE1) is a mitosis-specific protein that connects the AUGMIN complex to microtubules during spindle and phragmoplast formation. Phragmoplasts in *ede1* mutants are misformed, but no cytokinetic defects are observed (Lee et al., 2017). Surprisingly, the GAMMA-TUBULIN COMPLEX PROTEIN6 (GCP6) is not required for phragmoplast assembly (Miao et al., 2019). Gamma-tubulin ring complexes and AUGMIN complex proteins play both conserved and specific roles in microtubule nucleation during diffuse growth and cytokinesis. It will be interesting to identify how specificity is determined during specific cell-cycle stages.

## Microtubule severing proteins

The microtubule severing KATANIN (KTN) complex is composed of a p60 catalytic subunit and p80 microtubule-crossover-targeting subunits: both are required for proper microtubule severing in Arabidopsis (Bouquin et al., 2003; Wang et al., 2017a). Microtubule severing plays critical roles in microtubule array reorientation in response to intrinsic and extrinsic cues, including cell elongation and phragmoplast assembly and dynamics. Although mutants are viable, cell elongation and phragmoplast expansion rates are reduced in *ktn1* mutants. KTN1 is required for microtubule reorientation in response to mechanical force, suggesting that severing promotes microtubule reorientation (Bouquin et al., 2003; Wang et al., 2017a). KTN1 is also required for proper phragmoplast expansion, positioning, and morphology (Panteris et al., 2011; Komis et al., 2017; Ovečka et al., 2020; Panteris et al., 2021). *ktn1*, also called *fragile fiber2* (*fra2*) mutants have reduced callose accumulation and uneven cell plate formation (Panteris et al., 2021). KTN1 is thought to be recruited to the distal phragmoplast through interaction with the microtubule-binding protein MACET4/CORD4 to sever microtubules at the distal phragmoplast (Sasaki et al., 2019). Despite this, overexpression of MACET4/CORD4 in tobacco (*Nicotiana tabacum*) cultured cells did not lead to increased severing and its in vitro activity suggests it primarily nucleates microtubules (Schmidt and Smertenko, 2019). MACET4/CORD4 and KTN1 are both required for rapid phragmoplast expansion (Sasaki et al., 2019; Schmidt and Smertenko, 2019).

In the *ktn1* mutant, cell shape, microtubule orientation, and reorganization in response to mechanical cues are disrupted (Komis et al., 2017). Circumferential microtubule orientation propagated across several cell files around the ablation location in wild-type shoot apical meristem cells while in *ktn1* mutants circumferential orientation was limited to a single cell file (Uyttewaal et al., 2012). KTN1 is required for blue light-induced reorientation (Lindeboom et al., 2013). SPIRAL2/TORTIFOLIA1 is a plant-specific microtubule-binding protein required for promoting anisotropic growth and preventing organ twisting (Buschmann et al., 2004; Shoji et al., 2004). SPR2/TOR1 localizes to microtubule cross-overs and may block KTN1-mediated severing there (Wightman et al., 2013). An alternate or additional hypothesis is that SPR2/TOR1 stabilizes minus-ends to promote katanin-mediated severing (Nakamura et al., 2018). Various locations of SPR2 on microtubule-minus ends and microtubule crossovers may allow them to have opposite functions. Interestingly, AUGMIN reduction increases KTN1-mediated severing, suggesting that AUGMIN may also block KTN1 severing (Wang et al., 2018). Similarly, the microtubule bundling performed by MAP65-1 also prevents KTN1-mediated severing in vitro (Burkart and Dixit, 2019), within a time frame similar to induced cortical tension change (Colin et al., 2020). However, the mechanisms for mechanical force-dependent and blue light-induced microtubule reorientation are likely different.

## Microtubule bundling proteins with critical roles in diffuse growth and cytokinesis

Microtubule Organization1/Gemini1 (MOR1/GEM1) has an essential role in microtubule organization. *mor1* mutants disrupt diffuse growth and cytokinesis (Whittington et al., 2001; Twell et al., 2002; Kawamura et al., 2006). MOR1 contains conserved HEAT-repeat/TOG microtubule-binding domains (Whittington et al., 2001). MOR1 promotes microtubule bundling and polymerization and localizes to cortical microtubules, and mitotic microtubule arrays (Twell et al., 2002; Yasuhara et al., 2002; Hamada et al., 2004; Kawamura et al., 2006; Kawamura and Wasteneys, 2008). The temperature-sensitive *mor1* mutants have been used to clarify roles in regulating microtubule dynamics and aberrant cell plate formation (Eleftheriou et al., 2005; Kawamura and Wasteneys, 2008; Steiner et al., 2016). In addition to defects in diffuse growth, *mor1/gem1* mutants’ phragmoplasts sometimes split, leading to multiple aberrant new cell walls and irregular membrane accumulation in cell plates (Park and Twell, 2001; Eleftheriou et al., 2005). Interestingly despite the loss of parallel cortical microtubules and reduced microtubule polymer mass in the *mor1* mutant at restrictive temperature, cell wall crystallinity remained high, suggesting that microtubule mass is inversely related to cellulose crystallinity (Fujita et al., 2011).

A related TOG-domain-containing protein, CLIP-associating protein (CLASP), has a critical role in organizing interphase microtubules and promoting growth, does not have a conclusive role during cytokinesis (Ambrose et al., 2007; Halat et al., 2020; Kirik et al., 2007). CLASP, which preferentially localizes to cell edges, promotes microtubule passage across cell edges by preventing edge-induced microtubule depolymerization (Ambrose et al., 2011). This edge-induced microtubule depolymerization is similar to collision-induced catastrophe when the microtubule cross-over angles are >40° (Dixit and Cyr, 2004), highlighting the importance of both microtubule–microtubule and microtubule–cell edge interactions in controlling microtubule organization.

Another MAP family of proteins, called MAP65 because its founding member is 65 kilodaltons (Chang-Jie and Sonobe, 1993; Chan et al., 1999; Smertenko et al., 2000), has various roles in diffuse growth and cytokinesis. MAP65 family members typically bundle microtubules (Smertenko et al., 2004; Ho et al., 2012). MAP65-2 is required for axial growth in dark-grown hypocotyls: *map65-2* mutants have short hypocotyls. Generation of a double mutant with the related *map65-1* strongly reduces both light- and dark-grown hypocotyl elongation. The synthetic phenotype of the *map65-1 map65-2* double mutant indicates that these two proteins have overlapping roles in cell elongation (Lucas et al., 2011).

A few MAP65 family members play unique roles in phragmoplast organization during cytokinesis. The *map65-3/pleiade* mutant has defective cytokinesis because the two phragmoplast disks are spaced further apart, generating a wide phragmoplast midline (Söllner et al., 2002; Müller et al., 2004). MAP65-3 localizes to the phragmoplast midline and crosslinks antiparallel microtubules through its unique C-terminal domain. Other MAP65s do not rescue the *map65-3* mutant even when expressed with the *MAP65-3* promoter, supporting the unique role of MAP65-3 in phragmoplast organization (Ho et al., 2011a, 2012). Microtubule crosslinking is vital to accumulate microtubule plus-end directed EB1 particles and to restrict multiple plus-end directed kinesins in the midline, including several Kinesin 12 proteins (discussed in the “Plus-end-directed kinesins” section) (Ho et al., 2011a; Herrmann et al., 2018). MAP65-3, through multiple protein interactions, acts as a hub during cytokinesis. MAP65-3 interacts with POK2 and two closely related WD40 proteins, Budding Uninhibited by Benzimidazole (BUB) BUB3;1 and BUB3;2. Double *bub3;1 bub3;2* mutants grow normally. However, when *bub3;1 bub3;2* mutants are treated with caffeine, a drug known to disrupt phragmoplast attachment and maturation, MAP65-3 localization was abolished at the midline. Subsequent defects include phragmoplast expansion defects, short roots, and altered root cell morphology (Zhang et al., 2018). Interaction between MAP65-3 and its partners is required for their midline accumulation (Herrmann et al., 2018; Zhang et al., 2018). The localization and turnover of MAP65-3 from the phragmoplast midline in phosphoinositide kinase double mutants *pi4k-beta1 pi4k-beta2* is altered. This is consistent with the overstabilization of the phragmoplast, possibly due to an endocytosis defect (Lin et al., 2019). Restriction of the phragmoplast midline mediated by MAP65s in moss promotes timely vesicle fusion (Kosetsu et al., 2013; de Keijzer et al., 2017). Indeed, microtubule overlaps recruit the exocyst complex protein SEC6, which in turn recruits the vesicle fusion protein KEULE. SEC6 reduction slowed recruitment of KEULE to the midline (Tang et al., 2019). Overall, MAP65-3 performs critical functions during cytokinesis by crosslinking antiparallel microtubules, recruiting or retaining proteins at the phragmoplast midline often through direct interaction, and promoting vesicle fusion.

In addition to MAP65-3, MAP65-1 and MAP65-2 have partially redundant roles in cytokinesis revealed by mutant interactions with *map65-3. map65-1 map65-3* or *map65-2 map65-3* double mutants have synthetic cytokinesis defects (Sasabe et al., 2011). *map65-1 map65-2* double mutants do not have obvious cytokinesis defects although they produce fewer cells, suggesting they may still be involved in division (Lucas and Shaw, 2012). Interestingly, MAP65-1 localizes to the very leading edge of the phragmoplast, where it crosslinks antiparallel microtubules (Murata et al., 2013). Similarly, single *map65-4* mutants do not have an aberrant phenotype, but when combined with *map65-3* mutants, significant cytokinesis defects cause gametophyte lethality (Li et al., 2017a). MAP65-4 faintly localizes to the phragmoplast or phragmoplast midline (Smertenko et al., 2008). In the *map65-3* mutant, MAP65-4 localizes more prominently in the phragmoplast midline to partially replace MAP65-3 antiparallel bundling function (Li et al., 2017a). MAP65-4 bundles parallel and antiparallel microtubules, and promotes rescue in vitro (Fache et al., 2010). MAP65-4 also localizes to the division site but its function there is unknown (Li et al., 2017a). Further combinatorial mutant analysis of this important family of microtubule-bundling proteins will clarify unique and synergistic functions.

## Kinesins

Kinesins are mostly microtubule and sometimes also actin binding motor proteins that transport cargo including vesicles, organelles, and sometimes actin filaments or microtubules. Kinesins in plants either move toward the dynamic plus-end of microtubules or move toward the less dynamic minus-end of microtubules. The minus-end directed kinesins may functionally replace minus-end directed dyneins, which are rare in plants (Nebenführ and Dixit, 2018). Not surprisingly, kinesins are often required for cell expansion and/or cytokinesis.

The phragmoplast directs the movement of Golgi-derived vesicles, likely transported by plus-end directed kinesins, toward the newly developing cell plate. Indeed, even synthetic vesicles are efficiently transported to the newly developing cell plate (Esseling-Ozdoba et al., 2008). Although phragmoplast microtubules with attached vesicles have been observed by electron microscopy (Otegui et al., 2001), the specific kinesins have not been conclusively identified. However, a large number of kinesins play both essential and non-overlapping roles in cytokinesis and phragmoplast positioning, indicating the complexity in coordinating the cell plate formation (Nebenführ and Dixit, 2018; Smertenko et al., 2018). Many plus-end directed kinesins eventually accumulate in the phragmoplast midline, possibly reflecting the sheer abundance of microtubule plus-ends. It is likely that many plus-end directed kinesins transport vesicles containing both cell wall enzymes and precursors as well as proteins essential for regulating microtubule dynamics and vesicle fusion.

## Plus-end-directed kinesins

The KINESIN-4 family kinesins play various roles in cell elongation and cytokinesis (Kong et al., 2015), depending on the plant species. FRAGILE FIBER1 (FRA1) was initially identified in a forward genetic screen for deficiency in mechanical strength of inflorescence stems (Zhong et al., 2002). FRA1 is a highly processive plus-end directed kinesin (Zhu and Dixit, 2011). Reduced mechanical strength in *fra1* was attributed to defects in cellulose microfibril patterning (Zhong et al., 2002) but later findings demonstrate that *fra1-5* does not alter cellulose organization, motility, or density of CSCs at the plasma membrane (Zhu et al., 2015). While FRA1 does not affect cellulose synthesis directly, it associates with cellulose synthase microtubule-uncoupling proteins (CMUs), proteins that bind microtubules, localize to the cell plate, and prevent cell twisting (Zhu et al., 2015; Liu et al., 2016). By modulating the interaction between FRA1 and CMUs, lateral displacement of microtubules is regulated. Microtubule detachment from the cortex may affect the delivery of polysaccharides to the apoplast, contributing to the cell wall defects in *fra1-5* (Zhu and Dixit, 2011; Zhu et al., 2015; Ganguly et al., 2017; Ganguly et al., 2020). Indeed, *fra1-5* has reduced secretion of fucose-alkyne-labeled pectin (Zhu et al., 2015). However, *fra1-5* does not have any cytokinesis defects (Zhu et al., 2015). Similarly, Kinesin-4 in rice, BRITTLE CULM12, is required for proper cell wall composition and organization. Mutants have reduced cell number but lack obvious microtubule structural defects during cytokinesis (Zhang et al., 2010). By contrast, these proteins play a critical role in cytokinesis in moss: two closely related KINESIN-4s (Kin4-1a and Kin4-1c) localize to the phragmoplast midline (Miki et al., 2014) and minimize microtubule overlapping regions there (de Keijzer et al., 2017). KIN4-1c suppresses microtubule plus-end growth in vitro, so KIN4s may restrict microtubule overlap by inhibiting microtubule growth. Increased microtubule overlap regions accumulate more vesicles generating thick cell plates. Fascinatingly, the *kin4-1a kin4-1c* double mutant phragmoplast does not reorient, indicating division plane defects (de Keijzer et al., 2017).

Plus-end directed Kinesin-12 proteins participate in plant cell division, reviewed beautifully here (Müller and Livanos, 2019). PHRAGMOPLAST-ASSOCIATED KINESIN-RELATED PROTEIN1 (PAKRP1/KIN12A) and closely related PAKRP1-LIKE PROTEIN (PAKRP1L/KIN12B) have redundant roles in phragmoplast assembly in the male microspore. In the male microspore, callose accumulates in failed cell plates in the *pakrp1 pakrp1l* double mutants. However, vegetative cytokinesis is normal, indicating that there are additional, or different, proteins required for vegetative phragmoplast assembly (Lee et al., 2007). PAKRP1 and PAKRP1L interact with the ARM/HEAT repeats of the kinase TWO IN ONE (TIO), which is essential for cytokinesis in both microspores and during vegetative growth (Oh et al., 2005, 2012; Gillmor et al., 2016). These Kinesin-12 proteins are discussed here because their roles in specific divisions in the microspore and their interaction with TIO suggest that other plus-end directed kinesins may functionally rescue double mutants during vegetative growth, highlighting the need to generate higher-order mutant combinations or otherwise identify proteins that likely traffic vesicles essential for cytokinesis. Two other related Kinesin-12s PHRAGMOPLAST ORIENTING KINESIN1 (POK1) and POK2 also have redundant roles, but in phragmoplast positioning rather than cytokinesis (Müller et al., 2006). Both POK1 and POK2 localize to both the phragmoplast midline and the division site (Lipka et al., 2014; Herrmann et al., 2018; Mills et al., 2021). POK2 is required for fast phragmoplast expansion (Herrmann et al., 2018) and has diffusive plus-end directed movement (Chugh et al., 2018).

Several kinesin-7 plus-end directed kinesins are essential for cytokinesis. Two kinesin-7s, with M-phase specific activator (MSA) sequences, were called NACK1 and NACK2 in tobacco (Ito et al., 1998). Later, mutants in genes encoding NACK11/HINKEL and NACK2/TETRASPORE/STUD in tobacco and Arabidopsis and Nacka-c in moss were shown to have cytokinesis defects (Nishihama et al., 2002; Strompen et al., 2002; Yang et al., 2003; Naito and Goshima, 2015). HINKEL recruitment to the phragmoplast is partially dependent on RUNKEL, a microtubule-binding kinase-like protein essential for phragmoplast expansion (Krupnova et al., 2009, 2013). NACKs are processive plus-end directed kinesins, suggesting that they may transport cell plate building material (Naito and Goshima, 2015). HINKEL/NACK1 recruits Mitogen Activated Protein Kinase Kinase Kinase (MAPKKK) proteins to the phragmoplast midline through its C-terminal stalk domain (Sasabe and Machida, 2012; Sasabe et al., 2015). The Mitogen Activated Protein Kinase (MAPK) phosphorylates MAP65-1, MAP65-3, and EB1 proteins (Sasabe et al., 2006; Smertenko et al., 2006; Kohoutová et al., 2015). MAP65-1 phosphorylation, which also occurs by Aurora Kinase (Boruc et al., 2016), reduces microtubule binding and promotes proper microtubule disassembly at the phragmoplast lagging edge, which is critical for timely phragmoplast expansion (Sasabe et al., 2006; Smertenko et al., 2006). Like PAKRP1 and PAKRP1L, NACK2/TETRASPORE also interacts with the kinase TIO via the yeast-two-hybrid assay (Oh et al., 2014). Although another group of kinesin-7s are not essential for cytokinesis, they form a complex that promotes microtubule polymerization with a caspase-like protein essential for cytokinesis called Extra Spindle Poles1 (Moschou et al., 2013, 2016).

A land-plant specific “orphan” or ungrouped kinesin is sometimes required for cell elongation and is essential for phragmoplast microtubule interdigitation and cytokinesis in Arabidopsis, rice, and moss. The founding member, PHRAGMOPLAST ASSOCIATED KINESIN RELATED PROTEIN2 (PAKRP2), has a long neck and localizes to the phragmoplast midline and in phragmoplast-associated vesicles in Arabidopsis. PAKRP2 trafficking can be blocked by Brefeldin A (BFA) (Lee et al., 2001). PAKRP2 is a processive plus-end-directed kinesin that moves with an unusual combination of small step size and frequent steps to nearby filaments (Gicking et al., 2019). Processive movement and BFA sensitivity suggest it may transport Golgi-derived vesicles on the phragmoplast to the cell plate (Lee et al., 2001). In rice, the *PAKRP2* homolog, *STEMLESS DWARF1*, is essential for phragmoplast morphology and expansion during cytokinesis and also promotes cell elongation. Like PAKRP2, STEMLESS DWARF1 localizes to the phragmoplast midline (Fang et al., 2018). In moss, two PAKRP2-related kinesins called kinesin for interdigitated microtubules 1a (KINID1a) and KINID1b function similarly in both cell elongation and cytokinesis. *kinid1a kinid1b* double mutants have wide, frayed, and non-interdigitated phragmoplasts and reduced cell elongation due to a lack of microtubule bundling in the phragmoplast and at the growing tip, respectively. The double mutants also have chloroplast positioning defects, which may interfere with phragmoplast expansion and lead to cytokinesis defects (Hiwatashi et al., 2008, 2014).

## Minus end-directed kinesins

The minus-end-directed Kinesin-5 family plays a vital role in cytokinesis in moss, tobacco, and Arabidopsis but it may not participate directly in diffuse growth. A tobacco Kinesin-5, Kinesin-Related Protein (NtKRP), localizes to microtubules, and translocates antiparallel phragmoplast microtubules to minimize their overlap (Asada et al., 1997). The related Kinesin-5 *AtKRP125c* has a temperature-sensitive mutant, *radially swollen7* (*rsw7*), with spindle collapse, defective phragmoplast assembly, and cytokinesis defects. Although *rsw7* mutants have swollen cells suggesting defects in diffuse growth, and AtKRP125c localizes to cortical microtubules, the mutant phenotypes observed in mitosis and cytokinesis may account for apparent defects in cell shape (Bannigan et al., 2007). In moss, RNAi knockdown of all Kinesin-5 (a–d) homologs result in similar cytokinesis defects. Counterintuitively, Kinesin-5s are depleted from the phragmoplast midline via minus-end-directed movement (Miki et al., 2014). It is unclear how Kinesin-5 crosslinks or translocates antiparallel microtubules when it is excluded from antiparallel microtubule accumulation at the midline.

Several Kinesin-14 family minus-end-directed kinesins participate in cytokinesis. Kinesin-14s have diverse roles in spindle formation, chloroplast, and nuclear movement (Gicking et al., 2018). VARIED KERNEL SIZE1/ZmKIN11 (VKS1) is required for nuclear movement, spindle, and phragmoplast organization, with the *vks1* mutant leading to cytokinesis defects during endosperm cellularization, but not during vegetative development (Huang et al., 2019). This suggests other related proteins may functionally compensate during vegetative cytokinesis. A maize VKS1 paralog, KINDR, transports neocentromeres toward the spindle poles to promote meiotic drive, but has no role in vegetative cytokinesis (Dawe et al., 2018). Another Kinesin-14, kinesin with calponin homology (KCH, calponin homology domains suggest actin binding), binds both actin and microtubules, and localizes to mitotic microtubule structures (Xu et al., 2009; Buschmann et al., 2011; Klotz and Nick, 2012). The KCH protein KinG in Arabidopsis transports the transcription factor SHORTROOT, but *kinG* mutants have no growth or division defects, suggesting that other kinesins may perform redundant functions (Spiegelman et al., 2018). Mutants have tip growth defects in moss (Yamada and Goshima, 2018) and cell elongation defects in rice (Frey et al., 2010) due to organelle and nuclear movement defects. However, these proteins do have an obvious role in cytokinesis. A kinesin-like calmodulin binding protein (KCBP) (Reddy et al., 1996) localizes to the division site, but loss-of-function mutants do not have cytokinesis or phragmoplast positioning defects (Miki et al., 2014; Buschmann et al., 2015) but do have defects in polarized growth of the trichome (Oppenheimer et al., 1997; Tian et al., 2015). Microinjection of a calmodulin-domain-specific antibody that is thought to constitutively activate KCBP during anaphase causes aberrant phragmoplast positioning and assembly and subsequent cytokinesis defects (Vos et al., 2000). Targeted or conditional mutagenesis may provide more insight into KCBP’s role in cytokinesis. KCBP is a minus-end-directed kinesin, which after artificial oligomerization processively transports acidic phospholipid liposomes, that binds actin as well as microtubules in vitro, and is negatively regulated by calcium/calmodulin with clear roles in chromosome and organelle movement (Kao et al., 2000; Yamada et al., 2017; Yoshida et al., 2019).

## Cellulose synthase complexes

Cellulose is a major load-bearing component in the primary cell wall. It is composed of long chains of beta-1,4-linked glucans. Cellulose is made at the plasma membrane by a large complex named the CSC. The CSC was originally observed at the plasma membrane underlying microtubules using freeze fracture as a hexameric rosette structure (Mueller and Brown, 1980). More recently, live imaging was used to track in vivo movement of one of the CSC proteins CELLULOSE SYNTHASE 6 (CESA6) at the plasma membrane, where it often moved along microtubules (Paredez et al., 2006). The exact composition of the CSC in primary cell walls is unknown but likely contains a hexameric, equal stoichiometric combination of CESA1, CESA3 and either CESA6, CESA2, or CESA5 (Daras et al., 2009; Desprez et al., 2007; Persson et al., 2007; Wang et al., 2008; Gonneau et al., 2014; Hill et al., 2014). Mutants in cellulose synthase subunits produce tiny plants with cell elongation and sometimes cytokinesis defects. The *cesA1* mutant has defects in cytokinesis (Beeckman et al., 2002), while others such as *cesA6* may function only during cell expansion (Fagard et al., 2000), possibly due to *CESA6* redundancy with *CESA2* and *CESA5*.

Additional regulation of CESAs via post-translational modification such as phosphorylation may regulate stability or motility of CSCs (Li et al., 2014). Mutating putative phosphorylation sites in CESA1, CESA3, and CESA5 reduced their movement (Chen et al., 2010, 2016; Bischoff et al., 2011). Interestingly, the protein kinase BRASSINOSTEROID INSENSITIVE2 phosphorylates CESA1, highlighting an example of the hormonal regulation of cellulose synthesis via phosphorylation of CESA (Sánchez-Rodríguez et al., 2017).

Isoxaben is an herbicide that disrupts cellulose synthesis. Because many isoxaben-resistant mutants are mapped to CESAs, it is proposed that isoxaben may directly bind CESAs. However, the exact inhibition mechanism is unknown (Scheible et al., 2001; Desprez et al., 2002). Other chemicals, such as the cellulose biosynthesis inhibitor Endosidin 20, are useful tools to understand how CESA reaches the plasma membrane. Multiple *ces6* alleles were identified in a screen for mutants resistant to Endosidin20 (Huang et al., 2020). Transgenic lines carrying mutations in catalytic residues of cellulose synthase fail to localize to the plasma membrane, phenocopied by Endosidin20 treatment, highlighting Endosidin20 as a powerful tool to study CESA localization to the plasma membrane. Interestingly, although cellulose-deficient mutants or plants treated with isoxaben have growth defects, removing receptor-like kinases that mediate the cell wall integrity response partially restores growth without affecting cellulose levels (Hématy et al., 2007; Van der Does et al., 2017; Feng et al., 2018; Chaudhary et al., 2020). This suggests an intimate connection between the cell wall and cell-wall integrity pathways to limit growth under conditions disrupting the cell wall (Wolf et al., 2012). Altogether, herbicides that disrupt cellulose synthesis provide valuable tools for understanding assembly and trafficking of CESAs.

## Cellulose synthase-associated proteins

**Figure 2:**
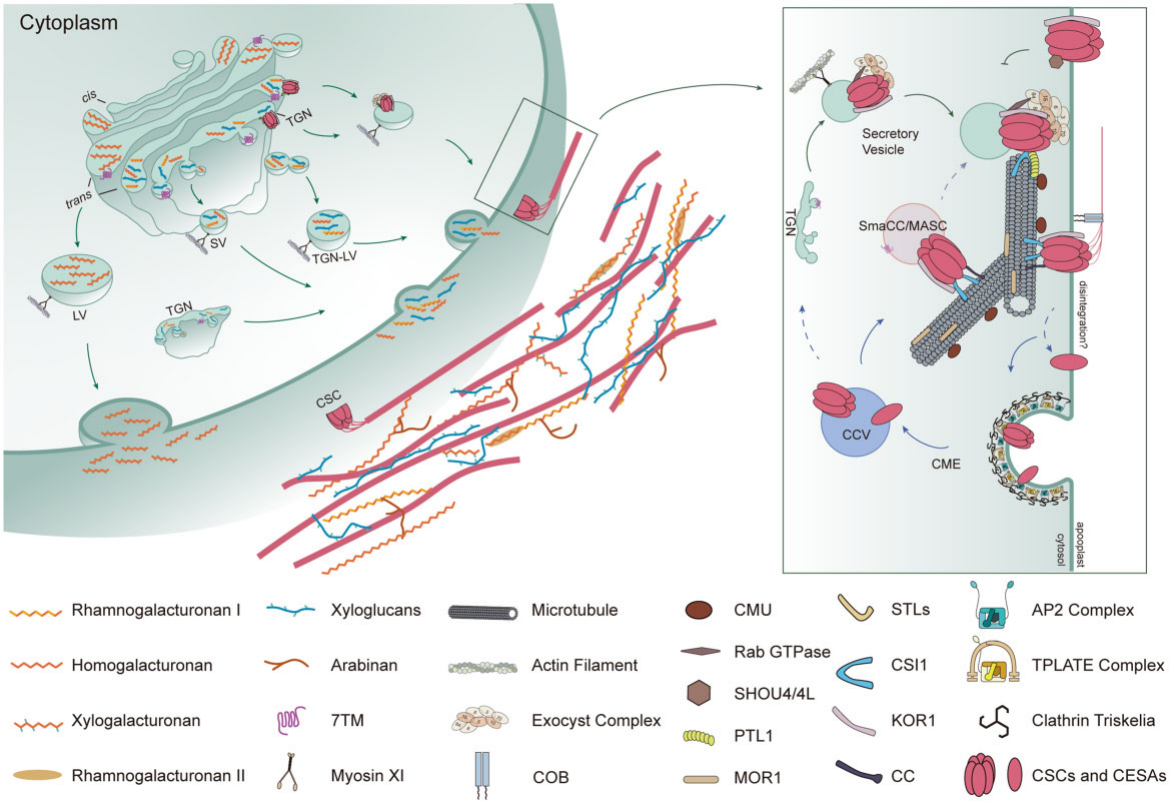
Model of trafficking of non-cellulosic polysaccharides and cellulose in the primary cell wall. The synthesis of non-cellulosic polysaccharides occurs in the Golgi. Non-cellulosic polysaccharides include xyloglucan and pectins. They are exported along actin filaments or microtubules in vesicles from the Golgi to the plasma membrane and incorporated into the apoplast. Cellulose is synthesized exclusively at the plasma membrane. The components involved in cellulose synthesis and its regulation are highlighted in the box on the right. Proteins associated with CSCs include Cellulose Synthase Interacting Protein 1 (CSI1), Companions of Cellulose synthase (CC), KORRIGAN1 (KOR1), and PATROL1 (PTL1). The accumulation of CSCs at the plasma membrane is influenced by exocytosis and endocytosis. Microtubules and actin filaments play important roles in the trafficking of CSCs to and from the membrane.

It was long proposed that the CSC interacts with microtubules at the plasma membrane by an elusive linker protein (Heath, 1974). The first linker protein identified, CELLULOSE SYNTHASE INTERACTIVE1 (CSI1, Figure 2), forms a physical association between active, plasma membrane-localized CSCs and cortical microtubules to establish and maintain organized and rapid cellulose biosynthesis (Gu et al., 2010; Bringmann et al., 2012; Li et al., 2012; Lei et al., 2013). The direct interaction between the central domain of CESA and CSI1 resides at multiple locations including N-, C-terminal and center regions of CSI1 while the interaction with microtubules are limited to the N- and C-terminal regions of CSI1 (Gu et al., 2010; Li et al., 2012; Lei et al., 2015). CESA particles fail to track with microtubules in the *csi1* mutant, and their velocity is reduced (Lei et al., 2012b). Two homologs have different expression levels: *CSI3* is expressed in meristematic zones while *CSI2* expression is not detected in all tissues. Single *csi3* mutants have no obvious aberrant phenotype but *csi1 csi3* double mutants have slower CESA particle movement, reduced cellulose content, and smaller dark-grown hypocotyls (Lei et al., 2013). It is currently unclear whether CSI1 or its homologs are essential during cytokinesis. Generating a triple mutant would be required to clarify whether they are required for cytokinesis.

There is mounting evidence that the regulation of cell growth is represented by a triangle that connects the cytoskeleton, cell wall, and cell shape. Microtubules guide the deposition of the cell wall and therefore determine the final shape of the cell. Cell geometry feedback influences the orientation of microtubules. CSCs are physically linked to microtubules via CSI1, highlighting the importance of the discovery of the key molecular linker underlying the parallel relationship between cellulose microfibrils and microtubules. However, despite the observation that CSC trajectories were uncoupled from the cortical microtubules in *csi1* null mutants (Li et al., 2012), cellulose microfibrils in the *csi1* mutants were oriented transversely instead of randomly (Xin et al., 2020). The transverse orientation of cellulose microfibrils is independent of CSC-microtubule linkage. However, the crossed-polylamellate wall architecture was lost in *csi1*. The loss of crossed-polylamellate wall was phenocopied by removing microtubules with the microtubule-depolymerizing drug oryzalin (Xin et al., 2020). These results are consistent with an earlier study showing that perturbation of microtubule rotation abolished the crossed-polylamellate wall texture (Chan et al., 2010). The crossed-polylamellate wall is important for restricting cell expansion laterally and promoting longitudinal expansion as loss of crossed-polylamellate wall coincides with loss of auxin-induced elongation (Xin et al., 2020).

Cellulase KORRIGAN1 (KOR1/GH9A1/RSW2/JIA) is the only known non-CESA protein that is an integral part of the CSC (Mansoori et al., 2014; Vain et al., 2014). KORRIGAN1 is an integral membrane endoglucanase first identified with an essential role in cell wall formation and cell elongation (Nicol et al., 1998). Later, a stronger mutant allele was identified that affected cytokinesis: KOR1 is also essential for cell plate formation (Lane et al., 2001). KOR1 protein localizes to multiple compartments including the Golgi, the TGN/early endosomes (EEs), and motile puncta at the plasma membrane (Robert et al., 2005; Lei et al., 2014), and the cell plate (Zuo et al., 2000). The transport of KOR1 to the cell plate requires two short amino acid motifs but these motifs are not required for localization at the plasma membrane (Zuo et al., 2000). As an integral part of the CSC, KOR1 may (1) remove disordered glucan chains during cellulose synthesis; (2) terminate cellulose synthesis; and/or (3) produce a short glucan chain “primer” for the initiation of cellulose synthesis. Although the exact role of KOR1 is unknown, glucanase activity is required for efficient cellulose biosynthesis (Nicol et al., 1998; Lane et al., 2001; Mansoori et al., 2014; Vain et al., 2014; Lei et al., 2014).

Non-CESA proteins that transiently associate with the CSC during cellulose biosynthesis include CSI proteins (described in the “Cellulose synthase-associated proteins” section) and COMPANION OF CELLULOSE SYNTHASE (CC) proteins. CC1 and 2 are two related plant-specific transmembrane domain-containing proteins that together prevent growth cessation after exposure to salt stress. *cc1 cc2* double mutants have short dark-grown hypocotyls with reduced cellulose content in growth medium supplemented with elevated salt. CC1 colocalizes with and interacts with CESA1, 3, and 6 (Endler et al., 2015, 2016). CC1 binds and bundles microtubules through four hydrophobic motifs that may promote microtubule reorganization after salt-stress (Kesten et al., 2019).

Additional proteins that are less well understood but important for cellulose synthesis and cell elongation include KOBITO, named for a Japanese word meaning small because the mutants are stunted, and COBRA (COB), named because the mutant roots had a snake-like shape (Schindelman et al., 2001; Pagant et al., 2002). *kobito* mutants have stunted growth, reduced cellulose accumulation, and broken cell walls (Pagant et al., 2002). KOBITO encodes a type-II membrane protein with confirmed localization at the plasma membrane. COB is a glycosyl–phosphatidyl inositol-anchored protein. COB localizes to the Golgi and cell wall (Roudier et al., 2005). *cob* mutants have disorganized cellulose microfibrils and reduced cellulose content (Roudier et al., 2005). In maize, *root-hairless3*, a monocot-specific *COB* homolog, promotes root hair growth and higher grain yield (Hochholdinger et al., 2008). *BRITTLE CULM1* (*BC1*) and *BC1-Like4* (*BC1L4*), *COB* homologs in rice, also promote cell expansion (Li et al., 2003; Dai et al., 2011). BC1 binds crystalline cellulose in vitro through an N-terminal domain and localizes to vesicles at the plasma membrane and in the cell wall (Liu et al., 2013). It is hypothesized that COB modulates cellulose assembly by its direct interaction with crystalline cellulose (Liu et al., 2013).

## The assembly and trafficking of CSC

The rosette CSCs were observed in the Golgi by electron microscopy, suggesting they are assembled there (Haigler and Brown, 1986). Consistently, immunogold labeling also showed CESA3 accumulation in the Golgi during cytokinesis (Zhang et al., 2016), but how CSCs assemble remains unknown. Two related Golgi-localized proteins named from the Greek word stello meaning “to set in order,” STELLO1 and STELLO1, are implicated in the assembly of CSCs because high molecular weight CSC complexes are reduced in *stello1 stello2* mutants (Zhang et al., 2016).

After the Golgi, it is unclear if trafficking to the plasma membrane is via Golgi-derived vesicles or the TGN/EE. One difficulty lies in the dual role of the TGN/EE: it contains both secretory and endocytosed material retrieved from the plasma membrane (Viotti et al., 2012; Sinclair et al., 2018; Renna and Brandizzi, 2020). Tracking fluorescent protein-tagged CESA movement revealed that CSC delivery coincides with Golgi pausing directly beneath the insertion site near microtubules (Crowell et al., 2009). However, another study showed partial colocalization between the CSC and TGN/EE markers (Gutierrez et al., 2009). Another type of CSC-containing vesicle, which accumulates under salt stress or isoxaben treatment, is referred to as small CESA compartments (SmaCCs) or microtubule-associated CESA compartments (MASCs). Because SmaCCs/MASCs periodically associate with the Golgi and TGN compartments, SmaCCs/MASCs might also deliver CSCs (Crowell et al., 2009; Gutierrez et al., 2009). However, a recent study showed that SmaCC/MASC formation depends on CSI1 and clathrin-mediated endocytosis (Bashline et al., 2013; Lei et al., 2015). SmaCCs/MASCs act in the rapid recycling of CSCs back to the plasma membrane after being relieved from high salt conditions. Prior to membrane fusion, a third type of CSC-containing vesicle co-localizes with the exocyst complex, an evolutionarily conserved vesicle tethering complex that regulates multiple membrane trafficking processes. CSI1 is a central hub for delivery of CSCs by regulating the tethering of CSC-containing vesicles along microtubules, by defining the domain in the plasma membrane for delivery, possibly by directly interacting with plasma membrane lipids via its C2 domain (Gu and Somerville, 2010), and by bridging multiple cellular components including the exocyst complex, PATROL1 (PTL1) and microtubules. Exocyst subunit mutants are defective in delivering CSCs to the plasma membrane (Zhu et al., 2018b). The regulation of CSC delivery also relies on a plant-specific protein PTL1 (Zhu et al., 2018b). PTL1 is a protein containing a Mammalian Uncoordinated13 Domain (MUN) that promotes exocyst vesicle fusion (Hashimoto-Sugimoto et al., 2013). PTL1 may prime the CSC-containing vesicles for plasma membrane fusion by the exocyst complex (Zhu et al., 2018b). PTL1 also accumulates at the early cell plate, but what it does there is not yet known (Maeda et al., 2020).

CSC transport to the cell plate likely occurs through three different mechanisms. First, CesA proteins (CESA1, CESA3, and CESA6) accumulate faintly on the phragmoplast and coalesce toward the cell plate as early as the tubular vesicular network, when Scarlet Pontamine staining suggested that cellulose accumulates (Miart et al., 2014). Cellulose is preferentially labeled with the Scarlet Pontamine dye (Anderson et al., 2010). Cellulose microfibrils are also observed either during or after cell plate fusion with the plasma membrane using field emission scanning electron microscopy (Fujita and Wasteneys, 2014). Second, when the phragmoplast disassembles in the middle of the cell, motile BFA-sensitive organelles, perhaps Golgi, transport CESA to the cell plate. Finally, when the cell plate fuses with the plasma membrane, CESAs move from the plasma membrane into the cell plate (Miart et al., 2014). Much remains to be discovered about how and when CESAs accumulate at the cell plate, and how or when they might interact with microtubules to initiate cellulose synthesis. Combined light and electron microscopy techniques will be essential for resolving these questions, discussed beautifully here (Chen et al., 2018).

While actin is not essential for formation or insertion of CSCs near microtubules, actin is required for the cell-wide distribution and movement of CSC-containing Golgi bodies and rapid CSC insertion (Gutierrez et al., 2009; Sampathkumar et al., 2013). The actin cytoskeleton provides tracks for myosin-based organelle movement, including the Golgi (Nebenführ and Dixit, 2018). CSC delivery and interaction with the exocyst complex also partially depend on the actin motor protein MYOSIN XI (Zhang et al., 2020). A direct connection between the actin cytoskeleton and exocyst complex was recently identified in moss with the exocyst protein SEC10 containing a formin domain (van Gisbergen et al., 2018).

CesA recruitment to the plasma membrane after isoxaben treatment depends partially on two related 7-transmembrane-domain (7TM) containing proteins with similarity to G-Protein Coupled Receptors (GPCRs) called 7TM1 and 7TM5. CSC secretion is affected in *7tm1 7tm5* double mutants under normal conditions even though mutants do not show any phenotype without isoxaben treatment. 7TM1 and 7TM5 localize to Golgi, TGN, and SmaCCs/MASCs, modulating CSC secretion after stress (McFarlane et al., 2021).

Two related novel transmembrane proteins SHOU4 and SHOU4L that directly interact with CESA1 and CESA3 may negatively regulate exocytosis of CSCs. The increased density of CSCs in *shou4 shou4l* double mutant epidermal cells presumably reflects increased rates of exocytotic CSC delivery. An elevated level of amorphous cellulose was detected within mutant inflorescence stems (Polko et al., 2018). It remains to be determined whether SHOU4-mediated CSC delivery occurs via a CSI1-dependent route, or represents a novel pathway.

The CSC is a large plasma membrane-localized complex that is predicted to be regulated via endocytosis (Lei et al., 2012a). During cytokinesis, clathrin-mediated endocytosis removes CESA particles both from the middle of the cell plate and the plasma membrane (Miart et al., 2014). The dynamin-like protein, DRP1A is required for clathrin-mediated endocytosis and cytokinesis, and colocalizes with clathrin light chain (Konopka et al., 2008; Konopka and Bednarek, 2008). The defects in *radially swollen9* (*rsw9*/*drp1a*) result in a cellulose deficiency and defective cell elongation and cytokinesis (Collings et al., 2008). A mutant locus in rice, *brittle culm 3*, mapped to a dynamin-related gene, *OsDRP2B*, which is likely required for clathrin-mediated endocytosis. The *brittle culm 3* mutant reduces rice cellulose synthase 4 (OsCESA4) protein accumulation at the plasma membrane, whereas overexpression of *OsDRP2B* has the opposite effect (Xiong et al., 2010). The direct evidence that CESA is a cargo protein in clathrin-mediated endocytosis came from the interaction between CESA and the medium subunit of ADAPTER PROTEIN COMPLEX 2 (AP2M) in vitro and by yeast-two hybrid assays. In *ap2m* knockout mutants, CESA particle density increases as a result of disrupted clathrin-mediated endocytosis (Bashline et al., 2013). TRANSDUCIN/WD40-2 (TWD40-2) is a member of the TPLATE complex, an evolutionarily ancient adaptor for clathrin-mediated endocytosis (Gadeyne et al., 2014). TPLATE is essential for growth and cytokinesis (Van Damme et al., 2006), while *twd40-2* knockdown also increases CESA particle density at the plasma membrane, indicating that the TPLATE complex regulates the endocytosis of CESA (Bashline et al., 2015). Similarly, the TPLATE Complex component MUNISCIN-LIKE (TML) and TPLATE interact with CESA6 and mediate the internalization of CESA6 from the plasma membrane (Sánchez-Rodríguez et al., 2018). Recent generation of a temperature sensitive *tplate* mutant will be a powerful tool to clarify its role in both diffuse growth and cytokinesis (Wang et al., 2021a).

## Non-cellulosic polysaccharide synthesis and delivery

Unlike cellulose, which is synthesized at the plasma membrane and in the late cell plate in the late stages of development, the synthesis of non-cellulosic polysaccharides occurs primarily in the Golgi (Camirand et al., 1987; Brummell et al., 1990; Gibeaut and Carpita, 1994). Major non-cellulosic polysaccharides of the primary cell wall including xyloglucan and pectin are discussed here. One important resource in identifying specific features of carbohydrates comes from a suite of well-characterized monoclonal antibodies (Pattathil et al., 2010). Xyloglucans are beta-1,4-linked glucans, similar to cellulose, with additional sugar modifications on branched side chains. Modification of xyloglucans, based on immunolocalization of terminal fucose antigens, likely occurs within the trans cisternae of the Golgi and the TGN (Zhang and Staehelin, 1992). Consistent with this hypothesis, oligosaccharide-mass-profiling of Golgi-enriched fractions revealed less substituted xyloglucans compared with the apoplastic cell wall fractions (Obel et al., 2009). In *phosphatidylinositol 4-kinase b1* and *b2* (*pi4kb1 pi4kb2*) mutants, because the vesicles were unusually large, xyloglucan transport is observed within secretory vesicles from trans Golgi to TGN and to the cell surface (Kang et al., 2011). However, xyloglucan-synthesizing enzymes such as xylosyltransferase (XT1) and galactosyltransferase (MUR3) are detected in cis and medial cisternae of Golgi using immunogold labeling (Chevalier et al., 2010). While it is possible that XT1 and MUR3 are mislocalized in cis and medial Golgi due to overexpression, further investigation will clarify whether the initiation of xyloglucan side chains occurs in early Golgi compartments.

In addition to accumulating at the plasma membrane through the Golgi, xyloglucan accumulates in cell plates. Xyloglucan cell plate accumulation occurs in many species and cell types, including Arabidopsis endosperm, maize root cells, and tobacco cultured cells (Moore and Staehelin, 1988; Otegui and Staehelin, 2000; Sonobe et al., 2000; Baluska et al., 2005). Xyloglucan is trafficked through peripheral Golgi, to vesicles that are likely transported on microtubules to the cell plate (Moore and Staehelin, 1988). Consistently, xyloglucan accumulates faintly on BY-2 phragmoplasts (Sonobe et al., 2000). BFA treatment produces xyloglucan-rich BFA bodies near cell-plate leading-edges in maize root cells (Baluska et al., 2005). The endoxyloglucan transferase protein, which modifies xyloglucans, co-localizes with the ER, the phragmoplast, and the cell plate (Yokoyama and Nishitani, 2001). Recently, an elegant approach combining SYNTAXIN OF PLANTS61 (SYP61)-enriched vesicle isolation and glycome profiling was developed (Wilkop et al., 2019). SYP61 is a syntaxin that co-localizes with a subset of the TGN, and plays a key role in trafficking to the plasma membrane (Sanderfoot et al., 2001; Li et al., 2017b). SYP61 vesicles contain both terminal fucose and galactosylated xyloglucans as well as pectins (described in more detail below). Interestingly, the galactosylated xyloglucan is also present in cell wall fractions, indicating that substituted xyloglucan may already be in its final form in SYP61 TGN-derived vesicles (Wilkop et al., 2019). It is unlikely that SYP61-derived vesicles carry all non-cellulosic polysaccharides from the Golgi to the apoplast. Adaptation of this approach using different GFP-tagged proteins in wild-type and mutant lines will aid the characterization of polysaccharide transport.

Several pectin-synthesizing and modifying enzymes localize in the Golgi, including galacturonosyltransferase (GAUT)1 and GAUT7, HG methyltransferases QUASIMODO2 (QUA2) and QUA3 (Goubet and Mohnen, 1999; Sterling et al., 2001; Mouille et al., 2007; Atmodjo et al., 2011; Miao et al., 2011; Anderson, 2015). Pectin synthesis likely begins in the cis Golgi cisternae. The addition of side chains and subsequent modification occur in late Golgi compartments. Antibodies recognizing pectin-specific epitopes show that rhamnogalacturonan-I (RG-I) modification occurs in trans Golgi and TGN in sycamore maple (*Acer pseudoplatanus*) cells and alfalfa (*Medicago sativa*) root border cells (Zhang and Staehelin, 1992; Wang et al., 2017b). Pectin methylesterases (PMEs) are transported to the cell plate directly through Golgi-derived vesicles, rather than through the TGN, because PME accumulation is BFA insensitive in tobacco (Wang et al., 2016). Rhamnogalacturonan-II (RGII) co-accumulates with callose at the cell plate (Zhou et al., 2017).

A comprehensive study using 39 monoclonal antibodies against pectin epitopes revealed that various degrees of substitution of pectin are enriched in SYP61-derived vesicles (Wilkop et al., 2019). SYP61 vesicles contain many proteins involved in cell wall biosynthesis, including ECHIDNA (ECH) and the YPT/RAB GTPase-interacting proteins (YIPs). ECH forms a TGN-localized complex with YIP and is required for pectin and xyloglucan secretion. In *yip4a yip4b* and *ech* mutants, pectin and xyloglucan are trapped in the intracellular space, presumably due to ineffective transport to the extracellular space (Boutté et al., 2013; Gendre et al., 2013; Renna et al., 2018). A group of secretory carrier membrane proteins (SCAMPs) localize to the TGN and in secretory vesicle clusters (SVCs) budding from the TGN (Toyooka et al., 2009). Methyl-esterified homogalacturonans, revealed by dual-immunogold labeling, are present in both the TGN and SVCs where SCAMPs were located. Whether SCAMPs are involved in pectin secretion is unknown. We are far from a complete picture of transport of non-cellulosic polysaccharides from specific Golgi compartments to the apoplast. For example, little is known about the localization and assembly of RG-II. Developing techniques that distinguish transport of complex non-cellulosic polysaccharides through specific routes remains a major challenge. Comprehensive localization of >80 glycosyltransferases among which >67 are for pectin and >14 are for xyloglucan biosynthesis and modification will be challenging but immensely informative.

Several orthologous cellulose synthase-like proteins, named *CELLULOSE SYNTHASE LIKE5* (*CSLD5*) in Arabidopsis, *CSLD4* in rice, and *CSLD1* in maize, are mitotically expressed and required for cytokinesis. Mutants have defects in cell wall expansion and also produce cell wall stubs indicative of cytokinesis defects (Li et al., 2009; Yin et al., 2011; Hunter et al., 2012; Yoo et al., 2012; Yoshikawa et al., 2013; Gu et al., 2016). CSLD5 localizes to punctate structures and accumulates in the cell plate (Gu et al., 2016). The cell wall material generated by this enzyme is unknown, although it may be mannans or xylans (Liepman et al., 2005; Bernal et al., 2007). Interestingly, the Arabidopsis *clsd5* cytokinesis defect is enhanced by combining *csld5* together with either the *csld2* or the *clsd3* mutant, even though *CSLD2* and *CSLD3* typically regulate root-hair growth (Yin et al., 2011; Gu et al., 2016). Unambiguously identifying which cell wall material is generated by these cellulose-synthase like proteins will provide valuable insight into their roles.

## Cell plate

Vesicles arrive between interdigitated phragmoplast microtubules (de Keijzer et al., 2017) or a region lacking ribosomes and microtubule overlap called the cell plate assembly matrix that surrounds the cell plate (Seguí-Simarro et al., 2004; McMichael and Bednarek, 2013). Vesicle trafficking during cell plate formation is mediated by vesicle tethering monomeric RAB-GTPases (Minamino and Ueda, 2019). RAB-GTPases including RAB-A2, RAB-A3, and RAB-E1 are essential for cytokinesis and transit through the Golgi and TGN to the cell plate (Chow et al., 2008; Mayers et al., 2017). Another RAB-GTPase, RAB-A5c accumulates in unique vesicles and occasionally with the TGN. RAB-A5c localizes to the cell plate and is essential for cytokinesis and also diffuse growth (Kirchhelle et al., 2016, 2019; Elliott et al., 2020). RAB-A1a-c may function redundantly: triple mutants are hypersensitive to the chemical Endosidin1 during cytokinesis (Qi and Zheng, 2013). Putative guanine exchange factors (GEFs) for RAB GTPase are also required for cell plate formation including STOMATAL CYTOKINESIS DEFECTIVE1 (SCD1), SCD2, and Transport Protein Particle II (TRAPPII) complex components (Falbel et al., 2003; Chow et al., 2008; Jaber et al., 2010; Thellmann et al., 2010; Qi et al., 2011; McMichael et al., 2013; Mayers et al., 2017; Kalde et al., 2019). Recently, TRAPP-Interacting Plant Protein (TRIPP) was identified as a plant-specific member of the highly conserved TRAPPII complex. TRIPP is required for cytokinesis and is trafficked from the TGN to the cell plate (Garcia et al., 2020). Fascinatingly, SCD1, SCD2, and TRAPPII members interact with exocyst proteins (described in the following paragraph), possibly coordinating early and late stages of vesicle trafficking during cell plate formation (Rybak et al., 2014; Mayers et al., 2017). Four related proteins, BIG1-4, that share sequence homology with ADP-ribosylation factor (ARF) GEFs are essential for rerouting vesicles to the cell plate to complete cytokinesis (Richter et al., 2014).

The exocyst complex, a heteromeric complex with eight different subunits (SEC3, SEC5, SEC6, SEC8, SEC10, SEC15, EXO70A1, and EXO84b), is required for vesicle tethering during cytokinesis. Multiple subunits localized to the early and post-cytokinetic cell plate. Exocyst component mutants have defects in initial cell plate assembly (Fendrych et al., 2010; Wu et al., 2013; Rawat et al., 2017; Tang et al., 2019; Brejšková et al., 2021). Once the vesicles reach the midplane, they undergo fusion, mediated by SNARE protein complexes containing the cytokinesis-specific syntaxin KNOLLE (Lauber et al., 1997; Lukowitz et al., 1996; Heese et al., 2001; Zhang et al., 2011a; El Kasmi et al., 2013; Park et al., 2018; Won and Kim, 2020). SNARE complexes are preassembled in the ER, then trafficked through the Golgi and TGN to the cell plate (Chow et al., 2008; Karnahl et al., 2017). KNOLLE is restricted to the cell plate by RAB GTPase-mediated endocytosis (Boutté et al., 2010) and activated by the SEC1/MUNC18 protein KEULE, which also interacts directly with SEC6 (Waizenegger et al., 2000; Assaad et al., 2001; Wu et al., 2013).

The fused vesicles form dumbbell-shaped structures that then coalesce into a tubular–vesicular network (Samuels et al., 1995; Otegui et al., 2001). Dynamin-related proteins (DRPs), large GTPases essential for both cell plate formation and endocytosis, encircle membranes at the cell plate (Gu and Verma, 1996; Kang et al., 2003). In Arabidopsis, overexpression of the essential cytokinetic dynamin, phragmoplastin, causes overaccumulation of callose, possibly due to phragmoplastin-callose synthase (CALS1) interaction (Hong et al., 2001). Many DRPs localize to the cell plate (McMichael and Bednarek, 2013). DRP1A interacts with the membrane tubulation protein SH3P2 (Ahn et al., 2017) to transform vesicles into tubules. Together DRP1A and DRP2B localize to the phragmoplast midline and are essential for endocytosis during cytokinesis (Fujimoto et al., 2008; Fujimoto and Tsutsumi, 2014; Ekanayake et al., 2021).

Mutants that disrupt sterol formation or modification often have defective cytokinesis. DRP1A and sterols co-localize in the cell plate: removal of either disrupts localization and cell plate formation, suggesting their mutual requirement for high-lipid order in the cell plate (Frescatada-Rosa et al., 2014). Two related sterol methyltransferases, SMT2 and SMT3, generate C24-ethyl sterols. The *smt2 smt3* double mutants have misoriented phragmoplasts and cell wall stubs, which are partially rescued by exogenous application of sterols (Nakamoto et al., 2015). Other mutants with defects in sterol synthesis or modification also have defects in cytokinesis, likely due to defective endocytosis, independent of a role in steroid hormone brassinosteroid biosynthesis (Willemsen et al., 2003; Schrick et al., 2000; Men et al., 2008; Carland et al., 2010; Wang et al., 2021b). Other lipids are required for proper cytokinesis, including very long chain fatty acids, sphingolipids, diacylglycerol, and phosphoinositides (Bach et al., 2011; Molino et al., 2014; Simon et al., 2016; Vermeer et al., 2017) reviewed beautifully here (Caillaud, 2019).

## Callose is mostly found in the cell plate

Callose, a prominent component in the cell plate (Drakakaki, 2015), is rarely represented in primary cell walls, except in plasmodesmata (Wu et al., 2018) or under stress or damage inducing conditions (Cui and Lee, 2016; Záveská Drábková and Honys, 2017). Callose is composed of beta-1,3-linked glucans unlike the beta-1,4-linked glucan chains found in xyloglucans and cellulose. Multiple callose synthases have both discrete and overlapping functions in symplastic transport through plasmodesmata (Wu et al., 2018), wounding and pathogen response (Ellinger and Voigt, 2014; Wang et al., 2021c), phloem development (Slewinski et al., 2012), and pollen surface patterning (Schneider et al., 2016). Mixtures of cellulose with high amounts of callose provide more elasticity in vitro (Abou-Saleh et al., 2018), which may be a critical structural feature during cell plate growth. Biophysical modeling suggests that callose polymerization may provide the spreading force required to promote flattening of the tubulo-vesicular network into the fenestrated sheet (Jawaid et al., 2020).

Callose is synthesized by large protein complexes containing callose synthase (Glucan-synthase-like) integral membrane proteins (Schneider et al., 2016). A callose synthase CalS1/GSL6 localizes to the cell plate (Hong et al., 2001). However, while two other callose synthases, GSL8/MASSUE and GSL10 are essential for cytokinesis, CalS1 is not (Saatian et al., 2018; Töller et al., 2008; Chen et al., 2009; Thiele et al., 2009). A clever approach to circumvent death of callose deposition defective mutants uses a chemical, Endosidin7 (ES7), that prevents callose accumulation (Drakakaki et al., 2011). ES7 prevents callose synthesis in vitro by partially blocking UDP-glucose incorporation into 1,3-linked glucans from Arabidopsis cell membrane extracts. Surprisingly, ES7 does not alter wound- or stress-induced callose accumulation (Park et al., 2014). The specific target of ES7 is unknown, but blocking callose synthesis with ES7 affects cytokinesis in both land plants and algae, suggesting that the target of ES7 is highly conserved (Park et al., 2014; Davis et al., 2020).

Callose synthase activity depends on the presence of calcium (Kauss, 1985; Amor et al., 1995). The ER may be a calcium source for callose synthases during cell plate formation: ER–cell plate association occurs in diverse plant lineages (Porter and Machado, 1960; Schmiedel et al., 1981; Otegui et al., 2001). Intense calcium accumulation in the cell plate has been observed via staining (Wick and Hepler, 1980; Schmiedel et al., 1981) while calcium sequestration disrupts cell plate formation in *Tradescantia virginiana* (Jürgens et al., 1994). The *P. patens sabre* mutant has defects in ER connections with the cell plate that are linked to both slow and aberrant callose accumulation, and eventual cytokinesis defects (Cheng and Bezanilla, 2021). One tempting hypothesis is that the slow callose accumulation in the *sabre* mutant may be due to aberrant calcium gradients near the cell plate due to altered ER connections.

## Conclusion

Cell wall modification and construction are dynamic processes during which secretion and endocytosis are tightly controlled. There are many open questions remaining such as how cellulose and non-cellulosic polymer insertion is coordinated, how non-cellulosic polymers are trafficked between the Golgi and plasma membrane and on which cytoskeletal tracks, and how the cytoskeleton and CSC activity are influenced by mechanical and environmental cues.

## Accession numbers

Provided in Supplemental Data Set S1.

## Supplemental data

**Supplemental Data Set S1.** Summary of the cytoskeletal and cell wall proteins required for diffuse growth and cytokinesis. Purple text indicates that proteins are involved in both diffuse growth and cytokinesis. Red text indicates that proteins are likely involved in only diffuse growth. Blue text indicates that proteins are likely involved only in cytokinesis.

## Supplementary Material

koab249_Supplementary_Data

## Notes

The authors responsible for distribution of materials integral to the findings presented in this article in accordance with the policy described in the Instructions for Authors (https://academic.oup.com/plcell) are Ying Gu (yug13@psu.edu) and Carolyn G. Rasmussen (crasmu@ucr.edu)
